# Single cell dynamics and nitrogen transformations in the chain forming diatom Chaetoceros affinis

**DOI:** 10.1038/s41396-023-01511-z

**Published:** 2023-09-18

**Authors:** Rickard Stenow, Elizabeth K. Robertson, Martin J. Whitehouse, Helle Ploug

**Affiliations:** 1 Department of Marine Sciences, University of Gothenburg, Box 461, SE 405 30 Gothenburg, Sweden; 2 Swedish Museum of Natural History, Box 50 007, SE 104 05 Stockholm, Sweden

**Keywords:** Microbial communities, Biochemistry, Microbial ecology, Stable isotope analysis, Microbial ecology

## Abstract

Colony formation in phytoplankton is often considered a disadvantage during nutrient limitation in aquatic systems. Using stable isotopic tracers combined with secondary ion mass spectrometry (SIMS), we unravel cell-specific activities of a chain-forming diatom and interactions with attached bacteria. The uptake of ^13^C-bicarbonate and^15^N-nitrate or ^15^N-ammonium was studied in *Chaetoceros affinis* during the stationary growth phase. Low cell-to-cell variance of ^13^C-bicarbonate and ^15^N-nitrate assimilation within diatom chains prevailed during the early stationary phase. Up to 5% of freshly assimilated ^13^C and ^15^N was detected in attached bacteria within 12 h and supported bacterial C- and N-growth rates up to 0.026 h^−1^. During the mid-stationary phase, diatom chain-length decreased and ^13^C and ^15^N-nitrate assimilation was significantly higher in solitary cells as compared to that in chain cells. During the late stationary phase, nitrate assimilation ceased and ammonium assimilation balanced C fixation. At this stage, we observed highly active cells neighboring inactive cells within the same chain. In N-limited regimes, bacterial remineralization of N and the short diffusion distance between neighbors in chains may support surviving cells. This combination of “microbial gardening” and nutrient transfer within diatom chains represents a strategy which challenges current paradigms of nutrient fluxes in plankton communities.

## Introduction

Diatoms are responsible for ca. 20% of global primary production [1], and often produce chain-like colonies in plankton communities. Colony formation in phytoplankton occurs across many classes and impacts both nutrient fluxes and grazing potential [2–5]. Diatom chains are often found when nitrate (NO_3_^−^) concentrations and turbulence are high during spring blooms and in upwelling regions [4]. Models, laboratory, and field studies have demonstrated that long chains benefit from increased nutrient fluxes from high ambient water turbulence compared to short chains or solitary cells [6–10]. However, chain formation is considered a disadvantage during stagnant conditions and nutrient limitation due to close proximity between cells leading to within-chain competition for nutrients diffusing to the chain compared to solitary cells [6]. Culture studies show decreased chain-length in the stationary phase when concentrations of NO_3_^−^ are low [11], which may be an adaptation to diffusion-limited nutrient availability [4].

Over a decade ago, secondary ion mass spectrometry (SIMS) was introduced to microbial ecology [12]. In combination with stable isotopic tracer experiments, SIMS can reveal cell specific biological activities: carbon (C), dissolved inorganic nitrogen (DIN) assimilation, and transfer in mixed field populations, within colonies, between phytoplankton, and attached bacteria [10, 13–18]. With this experimental tool, it was demonstrated that field populations of chain-forming diatoms, such as *Chaetoceros affinis* can contribute up to 20% of total primary production despite only representing a few percent of the phytoplankton biomass in late summer when DIN concentrations reach the nM range [19]. Primary production was mainly based on remineralized ammonium (NH_4_^+^) (ref. [19, 20]). Interactions between *Chaetoceros* and bacteria in the phycosphere, i.e., the boundary layer with concentration gradients of nutrients and solutes adjacent to the cell surface [21–23] resulted in NH_4_^+^ assimilation rates up to four times higher in diatoms than those explained by diffusion from the ambient water [19]. Hence, bacteria-algae interactions can lead to NH_4_^+^ production and assimilation within the phycosphere which alleviates diffusion-limited nitrogen (N) regimes in field communities. Transfer of diatom derived C and N to heterotopic bacteria attached directly to suspended diatoms [15] and to diatoms in aggregates [24] have been shown to be more efficient than to free-living bacteria. How these transfers quantitatively benefit living diatoms at a single cell level in chains as compared to solitary cells has to our knowledge not yet been investigated.

Several *Chaetoceros* species lack high affinity transporters for NO_3_^−^ (ref. [25, 26]) and the switch from NO_3_^−^ to NH_4_^+^ assimilation is a well-known adaptation to regenerated production during summer [27]. Culture studies have demonstrated that xenic diatom strains can survive longer than their axenic counterparts during the stationary phase, presumably due to bacterial remineralization of dead diatoms and release of NH_4_^+^ (ref. [27]). NH_4_^+^ is key in several processes during the stationary phase which are important for the diatom life cycle and their survival on a larger time scale. Sexual reproduction in centric diatoms which is essential to restoring cell size after numerous vegetative cell divisions can be triggered by NH_4_^+^ (ref. [28]). Formation of sinking diatom aggregates (containing *Chaetoceros*) [29] with microenvironments of NH_4_^+^ concentrations up to 100-fold higher than in the surrounding ambient water occurs after diatom blooms [10]. Such NH_4_^+^ microenvironments may promote sexual reproduction between different clones [30]. The high aggregate sinking velocities transport cells to sediments where they can survive as resting stages for decades up to millennia, in dark and anoxic conditions [31, 32]. Resting stages of the chain forming diatom, *Skeletonema marinoi*, assimilate NH_4_^+^ at low rates in such conditions [33].

Here, we investigated C and N dynamics of *C. affinis* and its associated bacteria at a single cell level to quantify changes in nutrient acquisition and chain length during two weeks of the stationary growth phase, and how *C. affinis* stimulates growth of attached bacteria which can benefit diatom survival on short and long time scales. We report the pattern of cell decay within chains and solitary cells and propose that microbially mediated nutrient transfer between neighboring cells in chains during the late stationary phase can be significant in N limited regimes.

## Method

### Culture conditions and growth

*C. affinis* (CCAP 1010/27, isolated by Di Prisco, 2008), selected from the Gothenburg University Marine Algal Culture Collection was grown in: N limited F/8 medium mixed from autoclaved sea water [34], at 16 °C, 26 PSU, 12:12 h light:dark (L:D) cycle 60 µmol photons m^−2^ s^−1^ measured using a Scalar PAR Irradiance Sensor (Biospherical instruments Inc. San Diego, CA USA) without mechanical aeration. An inoculum of cells was transferred to 1 L Duran bottles filled with 800 mL modified F/8 medium with 117 µM NO_3_^−^, rather than 220 µM NO_3_^−^ in standard F/8 medium [34], for a final concentration of 3000 cells mL^−1^ determined using a Sedgewick Rafter counting chamber (see Diatom abundance and chain-length). The dissolved inorganic carbon to nitrogen ratio (DIC:DIN) was estimated to 17, resulting in a likely N limited medium. Diatom growth was monitored by a Varioskan Flash multimode fluorescence plate-reader (ThermoScientific Waltham, MA USA) using 425 nm excitation and 680 nm detection wavelength [35]. Fluorescence per cell was calibrated against counting chamber observations over the experiment to estimate PON. Dissolved NO_x_^−^ (NO_2_^−^ + NO_3_^−^) concentration was monitored using colorimetric analysis by VCl_3_ (ref. [36]). A pilot study was conducted prior to reveal the growth dynamics of the diatom strain and identify when the cultures entered the stationary growth phase as described above (data not shown). The sampling times was selected using the plate reader data.

### Experimental setup and isotopic enrichment

We conducted isotopic tracer experiments at day 9 (early stationary phase), day 15 (mid stationary phase), and day 21 (late stationary phase). For each experiment, nine bottles were enriched with 2.6–12% ^15^N-NO_3_^−^ and 11–13% ^13^C-HCO_3_^-^ just before the light phase. Triplicates of labeled bottles and an unlabeled control bottle were destructively sampled at each time point (T0, after 12 h in light (L), and after 24 h, 12:12 h L:D), in each growth phase. Samples were taken for isotopic analysis at the single cell level (SIMS/nanoSIMS), bacterial enumeration (DAPI staining), isotopic analysis of particulate organic carbon, and nitrogen (POC and PON), dissolved inorganic nutrients (NO_x_^−^, NH_4_^+^, silicate, ortho-phosphate), and cell counts (fixation with Lugol’s solution). In the late stationary phase, nine additional flasks were enriched with 1.0–2.4% ^15^N-NH_4_^+^ and 12% ^13^C-HCO_3_^−^ then incubated in light. Triplicate bottles were sampled immediately (T0), after 1.5 h, and 4.15 h after tracer addition along with unlabeled control bottles.

### Diatom abundance and chain-length

Samples (1 mL) from each bottle was fixed with Lugol solution and stored at 4 °C until analysis. Cells were counted using a Sedgewick Rafter counting chamber, samples were diluted to achieve no more than 30 cells per square. All samples were transferred using an autoclaved pipette tip with the end cut off, to avoid breaking chains. Over 300 cells were enumerated and had their chain-length determined for each replicate (over 14,400 counted cells in total).

### Dissolved inorganic carbon and nutrients

DIC was determined using Apollo SciTech AS-C5 DIC analyzer, Newark, Delaware, USA, from 12 mL samples stored in exetainers, each sample was measured 3–5 times and calibrated against certified reference material (Scripps Institution of Oceanography of the University of California, USA, Batch #189). Spectrophotometric analyses of dissolved NO_x_^−^ (see above), silicate [37], and phosphate [38] was performed on 1 mL filtered (0.2 µm cellulose-acetate) samples from each time point. Total NH_4_^+^ concentrations at each time point were determined fluorometrically on 40 mL of filtered sample after 4.15 h in the light [39].

### Dissolved and particulate isotope analyses

Samples (10–100 mL) for POC and PON were filtered on to pre-combusted GF/F filters (Whatman, 0.7 µm), dried overnight (60 °C), exposed to 20% HCl fume in a desiccator for 24 h to remove carbonates, and packed in tin capsules (Elementar Analysensysteme GmbH) for analysis in an elemental analyzer coupled to an isotope ratio mass spectrometer (EA-IRMS; ANCA-GSL; Sercon Ltd., Crewe, UK). POC and PON assimilation during 12 h L and 12:12 h L:D was calculated by subtracting the T0 POC/PON form the values after 12 h and 24 h, respectively. POC and PON assimilation during 12 h D was calculated by subtracting 12 h L from 12:12 h L:D. Concentrations of ^15^NH_4_^+^, ^15^NO_2_^−^, and ^15^NO_3_^−^ were determined in all experiments. The ^15^N NH_4_^+^ pool was determined by conversion to N_2_ gas using alkaline hypobromite iodine [40] and measured on a gas chromatograph coupled to an IRMS (GC-IRMS; Thermo Delta V Plus) [41]. Any ^15^NO_2_^−^ was converted to N_2_ gas by reduction with sulfamic acid [42] and measured on GC-IRMS. The ^15^N-NO_3_^−^ pool was measured by first treating samples with sulfamic acid to remove any NO_2_^−^ followed by reduction of NO_3_^−^ to N_2_ with cadmium [42, 43] and measurement on GC-IRMS. No significant nitrification (production of ^15^NO_2_^−^ or ^15^NO_3_^−^ in incubations with ^15^NH_4_^+^) was detected (data not shown).

### Secondary ion mass spectrometry

Samples (5 mL) were fixed in PFA (Paraformaldehyde, Electron Microscopy Sciences), final concentration 1–2% and stored at 4 °C overnight. The sample was then filtered onto a TTTP (2 µm pore size) filter and rinsed with PBS buffer (10×, pH 7.4). Filters were stored dry at room temperature until analysis. N and C assimilation in individual cells were analyzed using a large-geometry Secondary Ion Mass Spectrometer IMS 1280 (CAMECA, Gennevillers, France) at the NordSIMS facility (Natural History Museum, Stockholm, Sweden). Sample filters were sputtered with a thin (ca. 5 nm) Au layer, 237 areas of interest were pre-sputtered over 80 × 80 µm for 180 s with a 10 nA Cs^+^ primary beam to remove the Au coating and penetrate the Si frustule. The primary beam was then reduced to ca. 50 pA, which corresponds to a spatial resolution of ca. 0.5 mm, and rastered over a 70 × 70 µm area for data acquisition. Secondary ions were steered onto the ion optic axis using the Dynamical Transfer Optical System and a mass resolving power of 12,000 M/ΔM was used to separate the CN^−^ species from potential interferences (e.g., ^11^B^16^O^−^ at nominal mass 27). The ion species ^12^C^14^N^−^, ^13^C^14^N^−^, and ^12^C^15^N^−^ were measured in peak hopping mode using a low-noise ion counting electron multiplier with an electronically gated 44 ns deadtime, each cycle comprising integration times of 1, 2, and 5 s respectively for a total of 60 cycles. The acquired ion images were processed using CAMECA’s Winimage2 software. Regions of interest (ROI) were selected from ^12^C^14^N^−^ counts (cts) after 12 h L (light) and 12:12 h L:D (light:dark) for all experiments (713 cells), within which the isotope ratios ^13^C/^12^C and ^15^N/^14^N were calculated, including on pixel correction for the detector deadtime. The natural background ratios were determined from the average ratios in the non-labeled controls (^15^N:^14^N ratio: 0.00366, ^13^C:^12^C ratio: 0.01091).

### Bacterial abundance and nanoSIMS

From each flask, triplicates 2 mL samples were fixed in 1–2% PFA as above. From each replicate, 250 µL was filtered onto a black 0.2 µm cyclopore filter. DAPI staining was preformed according to ref. [44]. Bacteria per diatom were counted (12,535 bacterial cells counted attached to 477 diatoms). Samples were cut from the same TTTP filters as used for SIMS analysis, gold sputtered, and analyzed using a NanoSIMS 50 L (CAMECA, Gennevillers, France) at the chemical imaging infrastructure (Astra Zeneca, Mölndal, Sweden). Diatom attached bacteria were analyzed by locating diatoms using the attached light microscope to select the region before sputtering using a primary 150 pA Cs^+^ beam for 75 s. Free living bacteria were located on the filter by using the live view of secondary electrons in the pre-sputtered area. The area was sampled if bacteria were observed as judged by an increase in ^12^C^14^N^−^ abundance during live view. An area of 30 × 30 µm was analyzed with 512 × 512 pixels resolution using a cesium beam with ~18 nA current for 1 ms per pixel for up to 15 planes. Each layer was manually observed for burn-through. ^12^C^13^C^−^, ^12^C^12^C^−^, ^12^C^14^N^−^, ^12^C^15^N^−^, ^28^Si^−^, ^32^S, and secondary electrons was measured. The mass resolution for ^13^C^12^C and ^13^C_2_ was >9000 M/ΔM, ^12^C^14^N was >8000 M/ΔM, and >2000 M/ΔM for ^12^C^15^N respectively. The ROIs for individual bacteria attached to *C. affinis* and free-living bacteria found on the filter were determined using drift corrected and accumulated ^12^C^14^N^−^ cts. The positions of diatoms were verified using ^28^Si cts and secondary electrons. The isotopic ratio of ^15^N:^14^N was calculated for ROIs by dividing ^12^C^15^N^−^ abundance with ^12^C^14^N^−^ abundance. The ^13^C:^12^C isotopic ratio was calculated according to Eq. 1 (ref. [45]).

1 $${\,\!}^{13}{{{{{\rm{C}}}}}}{:}^{12}{{{{{\rm{C}}}}}}\,{{{{{{\rm{IR}}}}}}}_{{bacteria}}=\frac{{\,\!}^{12}{{{{{{\rm{C}}}}}}}^{13}{{{{{\rm{C}}}}}}}{{\,\!}^{12}{C}^{12}C\times 2}$$

Bacterial ^13^C:^12^C and ^15^N:^14^N ratios were measured until a stable mean and standard error of the mean (SE) was established. Natural background ratios were determined from ratios in the non-labeled controls (^15^N:^14^N ratio: 0.00373, ^13^C:^12^C ratio: 0.01094).

### C and N assimilation and growth

The isotopic ratios from SIMS and nanoSIMS were used as IR_t1_ to calculate k, the C-specific C or N-specific N assimilation rate (h^−1^) against the control IR_t0_ (ref. 10):

2 $$k\left({h}^{-1}\right)=\frac{{{IR}}_{t1}-{{IR}}_{t0}}{{F}_{{ambient}}({t}_{1}-{t}_{0})}$$

where *F*_ambient_ is the isotopic labeled fraction of either N or C in the ambient water and IR is the isotopic ratio in either bacteria, diatoms, or POM during *t*_1_ (12 or 24 h, NH_4_^+^ was extrapolated from 4 h).

The assimilation during incubation was calculated according to:

3 $$a=k\times t\times M$$

where *a* was assimilation (g) and *M* is the C or N mass per cell (g). The time (*t*) was either 12, 24 h (^15^NO_3_^−^ experiments), or 4.15 h (^15^NH_4_^+^ experiments) to normalize incubation time and to compare NH_4_^+^ incubations against NO_3_^−^. C and N specific growth rates *GR*_*bacteria*_ (h^−1^) was calculated for bacteria, assuming exponential growth using:

4 $${{GR}}_{{bacteria}=}\frac{{k}_{{bacteria}}}{{{{{\mathrm{ln}}}}}(2)}$$

### Diatom volume and biomass estimation

Cell volume in each bottle was estimated as a cylinder from average cell length and width using:

5 $${V}_{{diatom}}=\frac{{{{{{\rm{\pi }}}}}}\times {l}_{{diatom}}\times {d}_{{diatom}}^{2}}{4}$$

*V* = diatom volume (µm^3^), l = length (µm) and d = diameter (µm) [46]. Cell specific C content was calculated:

6 $${C}_{{diatom}}=0.288\times {V}_{{diatom}}^{0.811}$$

*C*_diatom_ = 10^−12^ g C diatom^−1^ and *V* = total cell volume in µm^3^ (ref. [47]). N_diatom_ was estimated from *C*_diatom_ using the Redfield ratio [48].

### Bacterial volume and biomass estimation

Bacterial volume (µm^3^) was estimated assuming a rod-shape approximated as a cylinder with a half-sphere at each end:

7 $${V}_{{bacteria}}=\frac{4}{3}\times \pi \times {r}_{{bacteria}}^{3}+\pi \times {{l}_{{bacteria}}\times r}_{{bacteria}}^{2}$$

where *l*_bacteria_ is bacterial length and *r*_bacteria_ is bacterial radii (µm). C and N biomass (10^−15^ g bacteria^−1^) was calculated from [49].

8 $${C}_{{bacteria}}=197\, \times {V}_{{bacteria}}^{0.46}$$

9 $${N}_{{bacteria}}=39\times {V}_{{bacteria}}^{0.38}$$

where *C*_bacteria_ and *N*_bacteria_ is the bacterial C and N biomass per bacteria (10^−15^ g).

### Statistical analysis

We ensured that enough single cells were analyzed to achieve representative, stable mean values by plotting the cell-specific activity against number of cells analyzed [10]. Figures were produced using ggplot2 and the tidyverse package, error-bars represent SE [50]. Diatoms were considered active/enriched if their average C enrichment ratio minus two times the standard deviation was larger than the natural abundance ratio plus two times the standard deviation in the unlabeled control. Additionally, cells were visually inspected for bacterial remineralization. A chi-square test was used to investigate the number of active/inactive cells in chains and found as solitary cells. The level of significance was set to *p* ≤ 0.05. We compared the observed distributions of active and inactive cells in chains to expected values in chains, calculated using probability theory, each chain was considered a Bernoulli process. Observed frequency of active and inactive cells was used to predict the theoretical distributions of fully active chains, mixed, or fully inactive chains when randomly drafting cells for chains for each chain length. The expected probability of finding a fully active or in active chain (*P*) was calculated:

10 $$P={i}^{n}$$

where, *n* is the chain length and *i* are the probability of finding an inactive or active cell. The odds of mixed chains were calculated by subtracting the odds of fully active and inactive chains from 1.

## Results

### Diatom growth and dissolved nutrients

The *Chaetoceros* cultures reached the stationary phase after 9 days (Fig. S1). DIC was 890 ± 27 µM, 815 ± 22 µM, and 792 ± 7 µM in the early (9 d), mid (15 d), and late stationary phase (21 d), respectively (Table S1). Dissolved NO_3_^−^ decreased from, 42 ± 6 µM during the early stationary phase to 39 ± 9 µM and 28 ± 5 µM in the mid and late stationary phase. Simultaneously, dissolved NH_4_^+^ increased from 0.20 ± 0.02 µM (early) to 0.35 ± 0.04 µM (mid), and 1.59 ± 0.20 µM (late), representing 0.46%, 0.88%, and 5.37% of total DIN concentrations (Table S1). The silicate concentration decreased from 211 ± 28 µM to 194 ± 45 µM, and 139 ± 24 µM during the early, mid, and late stationary phase, respectively. Concentration of dissolved phosphate decreased from 8.9 ± 0.3 µM to 6.7 ± 1 µM and 8.0 ± 0.2 µM (Table S1).

### Cell-specific C- and N assimilation in diatoms

**Fig. 1:**
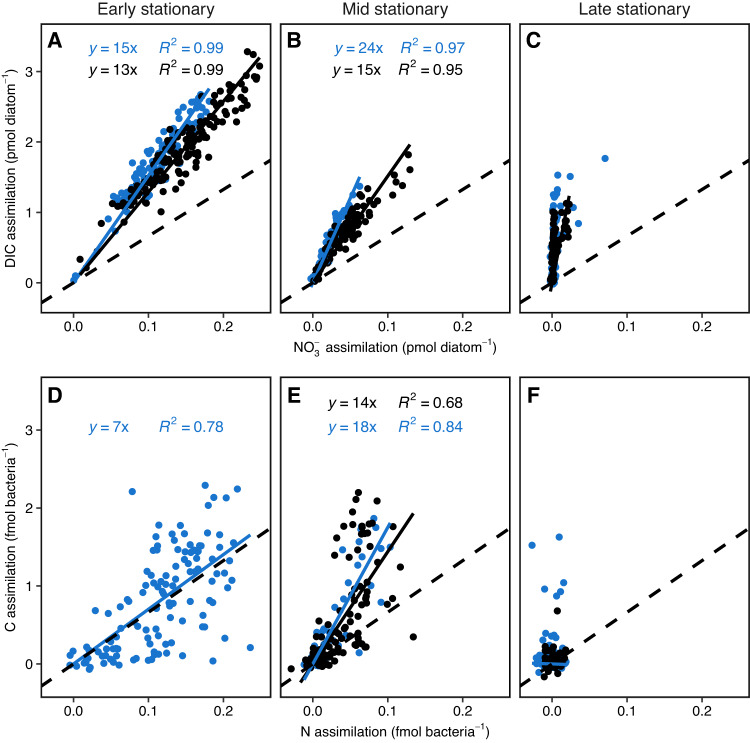
C and N assimilation by diatoms and transfers to their attached bacteria. **A**–**C** DIC and NO_3_^−^ assimilation in individual diatom cells, captured using SIMS, (**D**–**F**) Assimilation of freshly diatom derived C and N by individual diatom attached bacteria, captured using nanoSIMS. **A,** **D** Early stationary growth-phase, (**B**, **E**) mid stationary growth-phase and (**C**, **F**) late stationary growth-phase. Blue represents 12 h L and black 12:12 h L:D incubations. The dashed black line shows the Redfield C:N ratio and solid lines represent the observed C:N assimilation ratio. Note the different units between diatoms and bacteria (**A**–**C** and **D**–**F**).

**Fig. 2:**
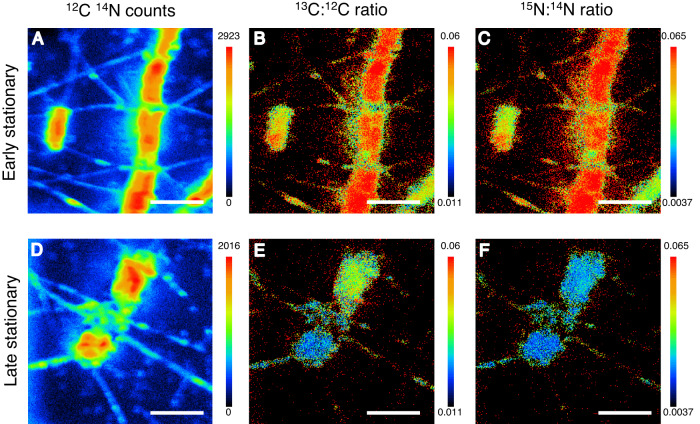
DIC and NO_3_^−^ assimilation in diatom chains and solitary cells captured using SIMS. **A**–**C** early stationary growth phase, **D**–**F** late stationary growth phase, (**A**, **D**) ^12^C^14^N ion counts per pixel, a proxy for diatom biomass used to determine the location of each cell. ^13^C:^12^C ratio, a proxy for DIC assimilation, note the higher variance between cells in subfigure (**E**) compared to (**B**). **C**, **F** ^15^N:^14^N ratio, a proxy for NO_3_^−^assimilation. Scalebar represents 20 µm.

Despite high ambient NO_3_^−^ concentrations, the average NO_3_^−^ assimilation in diatom cells decreased from 114 ± 4 fmol diatom^−1^ in the early stationary phase to 26 ± 2 fmol diatom^−1^, and 4.7 ± 1.2 fmol diatom^−1^ measured after 12 h L in the mid and late stationary phase, respectively (Fig. 1 and Table S2). The cell specific DIC:NO_3_^−^ assimilation ratio was 15.1, 25.2, and 97.9 in the early, mid, and late stationary phases, respectively, and thus significantly higher than Redfield ratio of 6.6 (Table S3). The diatoms continued to assimilate NO_3_^−^ during darkness at a low rate, but the overall C:N assimilation ratio after 12:12 h L:D cycle remained higher than the Redfield ratio. C and N assimilation did not result in net cellular growth during the mid and late stationary phase. No significant differences in diatom abundance between the early and the late stationary phase was determined by *t* test (27,000 ± 2200 diatom cells mL^−1^ and 28,000 ± 1900 diatom cells mL^−1^, *p* = 0.70) and cell size was stable (Tables S3 and S4). POC:PON mol ratio of the total biomass (diatoms and bacteria) was 7.0, 7.5, and 7.6 after 12:12 h L:D for the early, mid, and late stationary phases (Table S3), measured POC and PON per cell corresponded to values estimated from cell size (Table S5). In the early stationary phase, diatoms within the same chain showed similar C and NO_3_^−^ assimilation rates independent of position in the chain (Figs. 2, S2 and S3). However, C and NO_3_^−^ assimilation varied between chains, although the C:N ratio was stable within replicates. Chain-length decreased from 5.20 ± 0.13 diatom cells chain^−1^ in the early stationary phase to 2.56 ± 0.08 diatom cells chain^−1^ in the late stationary phase (Table S4), concurrent with increasing variability of C assimilation and a decrease in NO_3_^−^ assimilation rates within the same chain (Fig. 1 and Tables 1 and S6).

**Table 1:** Active and inactive diatom cells in chains and solitary cells during each growth phase, inactive cells were determined by visual inspection for bacterial remineralization or as having a 13C:12C ratio -SD × 2 smaller than that of the natural abundance + SD × 2.

Growth phase	Chain or solitary diatom	Active diatoms	Inactive diatoms	DIC assimilation active + inactive (pmol diatom^−1^)	DIC assimilation only active (pmol diatom^−1^)	NO_3_ assimilation active + inactive (pmol diatom^−1^)	NO_3_^−^ assimilation only active (pmol diatom^−1^)
Early stationary	In chain	180 (98.4%)	2 (1.6%)	1.83 ± 0.04	1.85 ± 0.04	0.132 ± 0.004	0.133 ± 0.003
Early stationary	Solitary cell	76 (96.2%)	3 (3.8%)	1.83 ± 0.06	1.88 ± 0.05	0.130 ± 0.005	0.134 ± 0.005
Mid stationary	In chain	103 (85.8%)	17 (14.2%)	0.64 ± 0.03	0.719 ± 0.03^a^	0.033 ± 0.002	0.037 ± 0.002^a^
Mid stationary	Solitary cell	30 (75.0%)	10 (25.0%)	0.70 ± 0.07	0.867 ± 0.06^a^	0.042 ± 0.005	0.054 ± 0.005^a^
Late stationary	In chain	82 (47.4%)	91 (52.6%)	0.50 ± 0.04	0.714 ± 0.04	0.005 ± 0.001	0.007 ± 0.001
Late stationary	Solitary cell	35 (55.6%)	28 (44.4%)	0.44 ± 0.05	0.741 ± 0.06	0.005 ± 0.001	0.008 ± 0.002

The percentages represent the proportion active and inactive cells for each growth phase. Average DIC and NO_3_^−^ ± SE, for all cells and only active cells.

^a^Indicates *p* < 0.05 calculated using a two tailed unpaired students *t* test between solitary cells and cell in chains for each growth phase.

### Bacteria-diatom interactions

Diatom attached bacteria increased during the stationary phase. Each diatom harbored on average 10 ± 0.4 bacteria diatom^−1^, 36 ± 4 bacteria diatom^−1^, and 86 ± 4 bacteria diatom^−1^ in the early, mid, and late stationary phase, respectively. The average exponential growth rate of attached bacteria was 0.0075 h^−1^ (Fig. S4 and Table S2). No autofluorescence was observed in the diatom attached bacteria, which were assumed to be heterotrophs, consuming dissolved organic matter, and releasing NH_4_^+^. This was supported by the observation that bacteria assimilated both C and N derived from diatoms close to Redfield ratio during the early stationary phase (Fig. 1). Diatom attached bacteria assimilated 0.82 ± 0.06 fmol freshly diatom-derived C bacteria^−1^, 0.45 ± 0.07 fmol C bacteria^−1^, and 0.11 ± 0.03 C bacteria^−1^ during the early, mid, and late stationary phase, after 12:12 h L:D. During the same period, diatom attached bacteria also assimilated 0.116 ± 0.005 fmol freshly diatom-derived N bacteria^−1^, 0.023 ± 0.004 fmol N bacteria^−1^, and 0.000 ± 0.001 fmol N bacteria^−1^ (Fig. 1, and Table S2). Free-living bacteria assimilated 0.17 ± 0.05 fmol freshly diatom-derived C bacteria^−1^, 0.13 ± 0.03 fmol C bacteria^−1^, and 0.07 ± 0.02 fmol C bacteria^−1^ during the early, mid, and late stationary phases after 12:12 h L:D and 0.084 ± 0.015 fmol freshly diatom derived N bacteria^−1^, 0.009 ± 0.002 fmol N bacteria^−1^, and −0.002 ± 0.001 fmol N bacteria^−1^. Hence, diatom attached bacteria assimilated up to 4.7 times more C during the early stationary phase and up to 2.6 times more N freshly derived from diatom DIC and DIN assimilation than their free-living counterparts during the mid-stationary phase. Up to 3.2 and 5.1% of freshly assimilated ^13^C and ^15^N was detected in attached bacteria within 12 h and supported bacterial C- and N- specific growth rates up to 0.026 h^−1^ and 0.008 h^−1^, respectively, close to the growth rate observed with DAPI of 0.0075 h^−1^ (Table S7).

### C and N dynamics in chains and solitary diatom cells

**Fig. 3:**
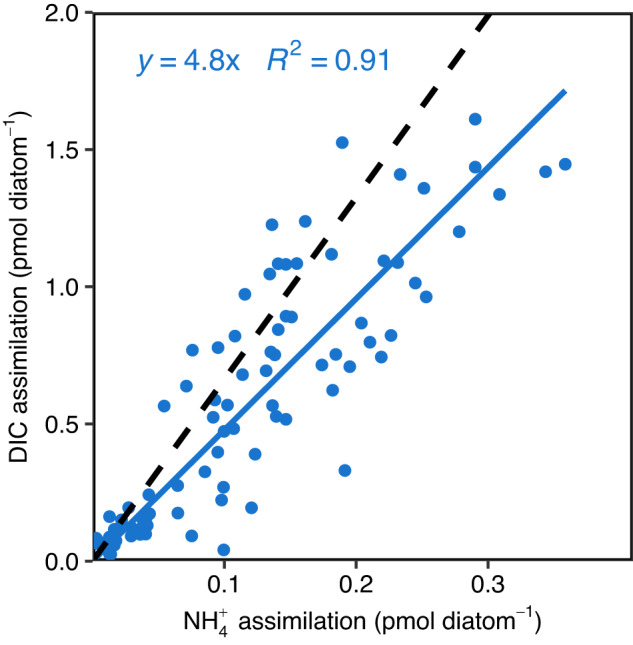
DIC and NH_4_^+^ assimilation by diatoms measured using SIMS extrapolated to 12 h L from 4.15 h L during the late stationary phase. The dashed line represents Redfield C:N ratio and the solid line represent the average observed C:N assimilation ratio.

Average DIC and NO_3_^−^ assimilation rates decreased simultaneously for both solitary cells and chain cells over the stationary phase (Fig. 1 and Table 1 and S6). On average, chain length decreased by 31% while C and NO_3_^−^ assimilation in chain cells decreased by 65 and 75%, from the early to the mid stationary phase respectively (Table S6). Hence, C and NO_3_^−^ assimilation in chain cells on average decreased comparatively more than chain length from the early to the mid stationary phase. However, statistical analysis of nutrient assimilation as a function of chain length under similar growth conditions was not feasible because short cell chains were rare in the early stationary phase, whereas long chains were rare in the mid and late stationary phase. Cell abundance remained relatively stable while chain length decreased (Tables S3, S4). Hence, long chains split into shorter chains during the stationary phase. Furthermore, cells with high C assimilation rates occurred frequently adjacent to inactive cells in the same chain during the late stationary phase (Figs. 2 and S3). The proportion of active chain cells declined from 98% in the early stationary phase to 47% in the late stationary phase. By comparison, the fraction of active solitary diatom cells declined from 96 to 56% during the same time (Table 1). The proportion of active/inactive cells within chains and solitary cells were not significantly different at any timepoint, as determined by a chi-square test (*p* = 0.53, 0.18, and 0.34) for the early mid and late stationary phase). The diatom cell-specific DIC, NO_3_^−^, and NH_4_^+^ assimilation rates were not significantly different between cells, within chains, and solitary cells due to high variances when active and inactive cells were pooled. However, both C and NO_3_^−^ assimilation by solitary cells were significantly higher as compared to cells in chains when only active cells were considered during the mid-stationary phase (Table 1). In the late stationary growth phase, bacterially regenerated NH_4_^+^ was the main N source for diatoms despite a low ambient concentration (5.4% of available DIN) compared to NO_3_^−^ (Table S1). The NH_4_^+^ assimilation rate was 110.5 ± 9.4 fmol diatom^−1^ and exceeded the NO_3_^−^ assimilation rates measured in the early stationary phase, contributing 96% of the diatoms DIN assimilation in the late stationary phase. The DIC:NH_4_^+^ assimilation ratio during the late stationary phase was close to the Redfield ratio (6.6) in contrast to the DIC:NO_3_^−^ assimilation ratio of 97.9 (Figs. 1 and 3).

### Distributions of active and inactive chain cells

Chain length decreased during the stationary phase leading to chains with two to four cells, only. Two-cell chains represented 33% of all cells in chains (58 of 173 observations). Of these two-cell chains, 51.7% (15 of 29 observations) were comprised of one active and one inactive cell while 24.1% (7 of 29 observations) were comprised of two active or inactive cells (Table 2). Three-cell chains represented 36.4% of all chain cells (63 of 173 observations) and represented the highest fraction of active cells (58.7 %). Of all three-cell chains, 75% contained both active and inactive cells. Four-cell chains represented 30% of the cells in chains (52 of 173 observations). One third (33.3%) of the four cell chains were completely inactive, and 66.7% of the chains contained a mixture of active and inactive cells (Table 2). The distributions of active and inactive cells in chains corresponded well to those predicted by probability theory for two- and three-cell chains. Four-cell chains had a higher portion of completely inactive chains than predicted and the number of inactive cells was significantly higher compared to the three-cell chains using a chi-square test (*p* = 0.005).

**Table 2:** Active and inactive diatoms in chains of varying length during the late stationary growth phase.

Chain length	Active cells	Inactive cells	Fully active chains	Mixed chains	Fully inactive chains	Expected fully active chains	Expected mixed chains	Expected fully inactive chains
Two-cell chain	29 (50%)	29 (50%)	7 (24.1%)	15 (51.7%)	7 (24.1%)	7 (22.5%)	14 (49.9%)	8 (27.7%)
Three-cell chain	37 (58.7%)	26 (41.3%)	3 (15.0%)	15 (75.0%)	2 (10.0%)	2 (10.6%)	16 (74.8%)	3 (14.6%)
Four-cell chain	16 (30.8%)	36 (69.2%)	0 (0.0%)	8 (66.7%)	4 (33.3%)	1 (5.0%)	11 (87.3%)	1 (7.7%)
Total	**82** (**47.4%)**	**91** (**52.6%)**						

The bold numbers are the sum of observations above and the relative frequency of active and inactive cells, used to calculate the probability of active and inactive cells in chains of different lengths.

Cells was checked for significant C assimilation (see method or Table 1 for more details) and visually for bacterial degradation. Expected values for chains were calculated using probability theory. The total chance for an active (47.4 %) or inactive cell (52.6 %) was used in n sequential biased coinflips to predict the chance of getting a fully active, mixed or fully inactive chain when randomly drafting cells for chains n cells long.

## Discussion

SIMS was first used in diatom ecology to quantify Si/C assimilation ratios in individual diatoms [51]. Recently, cell specific C and N assimilation have been analyzed with respect to chain length, cell position within long chains, and degree of ambient water turbulence in natural field populations [6, 10, 11]. Studies using nanoSIMS have focused on the microbial activities in the diatom phycosphere and in diatom aggregates [14, 15, 24]. Earlier studies demonstrated that both diatoms and bacteria can experience increased growth and survival during the exponential as well as the stationary phase during co-existence in cultures as compared to axenic diatom culture and bacterial mono cultures [27]. A host of synergistic interactions could explain this, see [22] for a review. Here, we quantitatively studied the dynamics and mutual benefits of interactions between diatoms and attached bacteria within diatom chains and solitary cells. We focused on the stationary phase characterized by complex diatom dynamics considering chain length and nutrients, and interactions with attached bacteria to gain a mechanistic understanding of the C and N exchange associated with diatom chains and solitary cells. To our knowledge, this is the first study to directly quantify cell-to-cell variations and dynamics of N assimilation and transfer within diatom chain cells as consequences of microbial activities.

### Variance of DIC and N assimilation within diatom chains

**Fig. 4:**
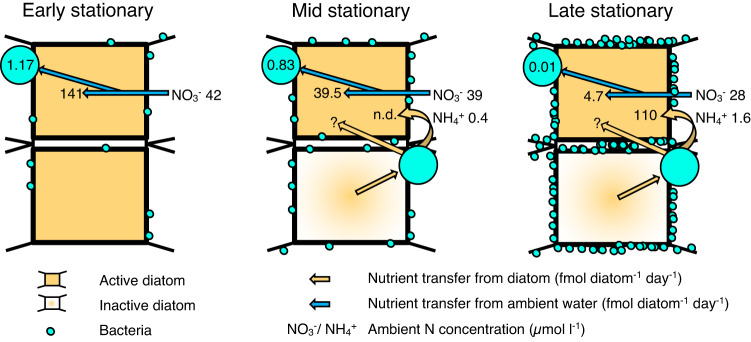
Measured N transfer between active diatoms, diatom attached bacteria, and inactive diatoms within a chain, during the early, mid and late stationary phase. Note how N assimilation shifts from NO_3_^−^ in the ambient water to NH_4_^+^ originating from bacterially remineralized neighboring diatoms between the growth phases. The question marks represent the ‘masked’ flux of N from a bacterially reminerialized diatom directly to the neighbor, before mixing with the ambient ^15^N-NH_4_^+^ enriched water. See Tables S1 and S2 for more details and SE.

Diatom chains form during cell division, when the silica frustule between daughter cells are fused together [52]. This results in chains consisting solely of clones. Chain length decreases if daughter cells with terminal setae are produced, which splits the chain during division [53, 54]. During mitosis, diatoms produce one daughter cell of the original cell size and one slightly smaller, leading to a decrease in average cell size. Cell volume can vary up to an order of magnitude between the largest and smallest cells of the same clonal strain [55]. Our study shows that while DIC and NO_3_^−^ assimilation rates varied between chains, the within-chain variation was low during the early stationary phase, likely due to the clonal nature of diatom chains. Cell-specific C and NO_3_^−^ assimilation rates varied 2- to 3-fold among all cells in chains during the early stationary phase similar to those previously reported for the chain forming diatom, *Skeletonema*, during the exponential phase [11]. During the mid and late stationary phases, we observed an increasing proportion of inactive diatoms both within chains and as solitary cells (Table 1). A significantly higher assimilation of both DIC and NO_3_^−^ occurred in solitary cells compared to cells in chains during the mid-stationary growth phase when only active cells were considered. Thus, the presence of inactive cells masked the signal of active cells. The majority of highly active cells in chains of all lengths (2–4 diatom cells chain^−1^) occurred in chains with at least one inactive cell during the late stationary phase, as predicted by probability theory (Table 2). In two-cell chains (Fig. 2), both cells have the same potential to assimilate nutrients from the ambient water according to diffusion theory. However, we observed that one cell was active while the other was inactive in over half of the chains. Such an imbalance rules out diffusion limitation from the ambient water in two-cell chains and may even suggest transfer of nutrients to active cells from neighboring inactive cells. Diffusion time of ions and solutes increases with the square of distance [56]. Owing to the short diffusion distance between neighboring chain cells, NH_4_^+^ remineralized by bacteria from dead diatoms may be efficiently transferred to living, neighboring cells by diffusion (Fig. S5). This NH_4_^+^ source would have had a natural ^15:14^N ratio. Our method therefore underestimates NH_4_^+^-based primary production by neighboring cells if this unlabeled NH_4_^+^ diffuses to a neighboring cell before mixing with the ^15^NH_4_^+^-enriched ambient water (Fig. 4). Our cultures had higher cell abundances (~30,000 cells mL^−1^) than in nature and the NH_4_^+^ produced from remineralized cells in the ambient water was high and could balance C fixation. In a low N regime with competing phytoplankton, the short diffusion distance between neighboring cells may significantly increase N transfer for surviving diatom cells in the same chain. It remains challenging to design experiments that directly measure these fluxes with methodologies available today due to the small spatial scales these fluxes operate on.

### Bacterial interactions

Diatoms supported substantial growth of attached and free-living bacteria by excreting freshly (<12 h old) assimilated and transformed DIC and DIN to the immediate environment (Table S2), presumably as dissolved organic matter. Such diatom-derived exudates can provide all nutrients required for bacterial growth [57]. Diatom attached bacteria clearly benefitted more from this release from their attachment directly to the source, as opposed to their free-living counterparts (Table S2). In return, diatoms could later balance their primary production with NH_4_^+^ assimilation from bacterial remineralization. The diatom DIC:NH_4_^+^ assimilation ratio was 4.8 during 12 h L (Fig. 3). Thus, bacteria supported diatoms with remineralized NH_4_^+^ at a rate which was close to that of NO_3_^−^ assimilation during the early stationary phase, as previously suggested by Grossart [27]. In the early stationary phase, nutrients transferred to attached bacteria did not exceed 0.44 and 0.83% of total diatom C and N assimilation, respectively (Table S2). Thus, “microbial gardening” of bacteria serves quantitatively as a low-cost solution to diatoms as an alternative NH_4_^+^ source to support living diatoms and completion of the life cycle after the late stationary phase (see ‘introduction’). NH_4_^+^ production from the random distribution of decay within diatom chains offers a previously overlooked nutrient source for surviving cells in the same chain as well as a mechanism to reduce the number of clones before sexual reproduction. Hence, chain formation and microbial interactions support both short term and long survival in diatoms.

### Supplementary information

SUP

## Data Availability

The datasets generated during and analyzed during the current study are available from the corresponding author upon reasonable request.

## References

[1] Nelson DM, Tréguer P, Brzezinski MA, Leynaert A, Quéguiner B (1995). Production and dissolution of biogenic silica in the ocean: revised global estimates, comparison with regional data and relationship to biogenic sedimentation. Glob Biogeochem Cycles.

[2] Ploug H, Stolte W, Jørgensen BB (1999). Diffusive boundary layers of the colony-forming plankton alga *Phaeocystis sp*.- implications for nutrient uptake and cellular growth. Limnol Oceanogr.

[3] Pančić M, Kiørboe T (2018). Phytoplankton defence mechanisms: traits and trade-offs. Biol Rev.

[4] Kenitz KM, Orenstein EC, Roberts PLD, Franks PJS, Jaffe JS, Carter ML (2020). Environmental drivers of population variability in colony‐forming marine diatoms. Limnol Oceanogr.

[5] Selander E, Jakobsen HH, Lombard F, Kiørboe T (2011). Grazer cues induce stealth behavior in marine dinoflagellates. Proc Natl Acad Sci USA.

[6] Pahlow M, Riebesell U, Wolf-Gladrow DA (1997). Impact of cell shape and chain formation on nutrient acquisition by marine diatoms. Limnol Oceanogr.

[7] Karp-Boss L, Boss E, Jumars PA (1996). Nutrient fluxes to planktonic osmotrophs in the presence of fluid motion. Oceanogr Mar Biol Annu Rev.

[8] Musielak M, Karp-Bboss L, Junars PA, Fauci LJ (2009). Nutrient transport and acquisition by diatom chains in a moving fluid. J Fluid Mech.

[9] Dell’Aquila G, Ferrante MI, Gherardi M, Cosentino Lagomarsino M, Ribera d’Alcalà M, Iudicone D (2017). Nutrient consumption and chain tuning in diatoms exposed to storm-like turbulence. Sci Rep..

[10] Bergkvist J, Klawonn I, Whitehouse MJ, Lavik G, Brüchert V, Ploug H (2018). Turbulence simultaneously stimulates small- and large-scale CO_2_ sequestration by chain-forming diatoms in the sea. Nat Commun.

[11] Olofsson M, Kourtchenko O, Zetsche E-M, Marchant HK, Whitehouse MJ, Godhe A (2019). High single-cell diversity in carbon and nitrogen assimilations by a chain-forming diatom across a century. Environ Microbiol.

[12] Musat N, Halm H, Winterholler B, Hoppe P, Peduzzi S, Hillion F (2008). A single-cell view on the ecophysiology of anaerobic phototrophic bacteria. Proc Natl Acad Sci USA.

[13] Adam B, Klawonn I, Svedén JB, Bergkvist J, Nahar N, Walve J (2016). N^2^-fixation, ammonium release and N-transfer to the microbial and classical food web within a plankton community. ISME J.

[14] Arandia-Gorostidi N, Weber PK, Alonso-Sáez L, Morán XAG, Mayali X (2017). Elevated temperature increases carbon and nitrogen fluxes between phytoplankton and heterotrophic bacteria through physical attachment. ISME J.

[15] Arandia‐Gorostidi N, Alonso‐Sáez L, Stryhanyuk H, Richnow HH, Morán XAG, Musat N (2020). Warming the phycosphere: differential effect of temperature on the use of diatom‐derived carbon by two copiotrophic bacterial taxa. Environ Microbiol.

[16] Schoffelen NJ, Mohr W, Ferdelman TG, Duerschlag J, Littmann S, Ploug H (2019). Phosphate availability affects fixed nitrogen transfer from diazotrophs to their epibionts. ISME J.

[17] Klawonn I, Van den Wyngaert S, Parada AE, Arandia-Gorostidi N, Whitehouse MJ, Grossart H-P (2021). Characterizing the “fungal shunt”: parasitic fungi on diatoms affect carbon flow and bacterial communities in aquatic microbial food webs. Proc Natl Acad Sci.

[18] Mayali X (2020). NanoSIMS: microscale quantification of biogeochemical activity with large-scale impacts. Ann Rev Mar Sci.

[19] Olofsson M, Robertson EK, Edler L, Arneborg L, Whitehouse MJ, Ploug H (2019). Nitrate and ammonium fluxes to diatoms and dinoflagellates at a single cell level in mixed field communities in the sea. Sci Rep..

[20] Klawonn I, Bonaglia S, Whitehouse MJ, Littmann S, Tienken D, Kuypers MMM (2019). Untangling hidden nutrient dynamics: rapid ammonium cycling and single-cell ammonium assimilation in marine plankton communities. ISME J.

[21] Cole JJ (1982). Interactions between bacteria and algae in aquatic ecosystems. Annu Rev Ecol Syst.

[22] Amin SA, Parker MS, Armbrust EV (2012). Interactions between diatoms and bacteria. Microbiol Mol Biol Rev.

[23] Seymour JR, Amin SA, Raina J-B, Stocker R (2017). Zooming in on the phycosphere: the ecological interface for phytoplankton-bacteria relationships. Nat Microbiol.

[24] Arandia-Gorostidi N, Berthelot H, Calabrese F, Stryhanyuk H, Klawonn I, Iversen M (2022). Efficient carbon and nitrogen transfer from marine diatom aggregates to colonizing bacterial groups. Sci Rep..

[25] Santin A, Caputi L, Longo A, Chiurazzi M, Ribera d’Alcalà M, Russo MT (2021). Integrative omics identification, evolutionary and structural analysis of low affinity nitrate transporters in diatoms, diNPFs. Open Biol.

[26] Pelusi A, Ambrosino L, Miralto M, Chiusano ML, Rogato A, Ferrante MI (2023). Gene expression during the formation of resting spores induced by nitrogen starvation in the marine diatom *Chaetoceros socialis*. BMC Genom.

[27] Grossart H-P (1999). Interactions between marine bacteria and axenic various conditions in the lab. Aquat Micro Ecol.

[28] Moore ER, Bullington BS, Weisberg AJ, Jiang Y, Chang J, Halsey KH (2017). Morphological and transcriptomic evidence for ammonium induction of sexual reproduction in *Thalassiosira pseudonana* and other centric diatoms. PLoS One.

[29] Gotschalk CC, Alldredge AL (1989). Enhanced primary production and nutrient regeneration within aggregated marine diatoms. Mar Biol.

[30] Behrenfeld MJ, Halsey KH, Boss E, Karp‐Boss L, Milligan AJ, Peers G (2021). Thoughts on the evolution and ecological niche of diatoms. Ecol Monogr.

[31] Härnström K, Ellegaard M, Andersen TJ, Godhe A (2011). Hundred years of genetic structure in a sediment revived diatom population. Proc Natl Acad Sci USA.

[32] Sanyal A, Larsson J, Wirdum F, Andrén T, Moros M, Lönn M (2022). Not dead yet: diatom resting spores can survive in nature for several millennia. Am J Bot.

[33] Stenow R, Olofsson M, Robertson EK, Kourtchenko O, Whitehouse MJ, Ploug H (2020). Resting stages of *Skeletonema marinoi* assimilate nitrogen from the ambient environment under dark, anoxic conditions. J Phycol.

[34] Guillard RRL (1975). Culture of marine invertebrate animals. culture of marine invertebrate animals.

[35] Gross S, Kourtchenko O, Rajala T, Andersson B, Fernandez L, Blomberg A (2018). Optimization of a high-throughput phenotyping method for chain-forming phytoplankton species. Limnol Oceanogr Methods.

[36] Schnetger B, Lehners C (2014). Determination of nitrate plus nitrite in small volume marine water samples using vanadium(III)chloride as a reduction agent. Mar Chem.

[37] Grashoff K, Kremling K, Ehrhard M (1999). Methods of seawater analysis. 3rd completely revised and extended edition.

[38] Strickland JHD, Parsons TR. A practical handbook of seawater analysis. Ottawa: Fisheries Research Board of Canada, Bulletin; 1972.

[39] Holmes RM, Aminot A, Kérouel R, Hooker BA, Peterson BJ (1999). A simple and precise method for measuring ammonium in marine and freshwater ecosystems. Can J Fish Aquat Sci.

[40] Risgaard-Petersen N, Rysgaard S, Revsbech NP (1995). Combined Microdiffusion-Hypobromite oxidation method for determining Nitrogen-^15^ isotope in ammonium. Soil Sci Soc Am.

[41] Dalsgaard T, De Brabandere L, Hall POJ (2013). Denitrification in the water column of the central Baltic Sea. Geochim Cosmochim Acta.

[42] Füssel J, Lam P, Lavik G, Jensen MM, Holtappels M, Günter M (2012). Nitrite oxidation in the Namibian oxygen minimum zone. ISME J.

[43] McIlvin MR, Altabet MA (2005). Chemical conversion of nitrate and nitrite to nitrous oxide for nitrogen and oxygen isotopic analysis in freshwater and seawater. Anal Chem.

[44] Porter KG, Feig YS (1980). The use of DAPI for identifying aquatic microfloral. Limnol Oceanogr.

[45] Pett-Ridge J, Weber PK. NanoSIP: NanoSIMS Applications for Microbial Biology. Methods Mol Biol. 2012;881:375–408. 10.1007/978-1-61779-827-6_13 22639220

[46] Sun J (2003). Geometric models for calculating cell biovolume and surface area for phytoplankton. J Plankton Res.

[47] Menden-Deuer S, Lessard EJ (2000). Carbon to volume relationships for dinoflagellates, diatoms, and other protist plankton. Limnol Oceanogr.

[48] Redfield AC. On the proportions of organic derivatives in sea water and their relation to the composition of plankton. In: Daniel RJ (ed). James Johnstone memorial volume. Liverpool: The University Press of Liverpool; 1934. pp 176–92.

[49] Khachikyan A, Milucka J, Littmann S, Ahmerkamp S, Meador T, Könneke M (2019). Direct Cell Mass Measurements Expand the Role of Small Microorganisms in Nature. Appl Environ Microbiol.

[50] Wickham H, Averick M, Bryan J, Chang W, McGowan L, François R (2019). Welcome to the Tidyverse. J Open Source Softw.

[51] Mejía LM, Isensee K, Méndez-Vicente A, Pisonero J, Shimizu N, González C (2013). B content and Si/C ratios from cultured diatoms (*Thalassiosira pseudonana* and *Thalassiosira weissflogii*): relationship to seawater pH and diatom carbon acquisition. Geochim Cosmochim Acta.

[52] Fryxell GA (1978). Chain-forming diatoms: three species of *Chaetoceracae*. J Phycol.

[53] Gherardi M, Amato A, Bouly J-P, Cheminant S, Ferrante MI, D’Alcalá MR (2016). Regulation of chain length in two diatoms as a growth-fragmentation process. Phys Rev E.

[54] Amato A, Dell’Aquila G, Musacchia F, Annunziata R, Ugarte A, Maillet N (2017). Marine diatoms change their gene expression profile when exposed to microscale turbulence under nutrient replete conditions. Sci Rep..

[55] Assmy P, Hernández-Becerril DU, Montresor M (2008). Morphological variability and life cycle traits of the type species of the diatom genus *Chaetoceros, C. diahaeta*. J Phycol.

[56] Berg, H C. Random walks in biology. Princeton: Princeton University Press; 1993.

[57] Eigemann F, Rahav E, Grossart H, Aharonovich D, Sher D, Vogts A (2022). Phytoplankton exudates provide full nutrition to a subset of accompanying heterotrophic bacteria via carbon, nitrogen and phosphorus allocation. Environ Microbiol.

