# Supplementary material for: Single cell dynamics and nitrogen transformations in the chain forming diatom Chaetoceros affinis
Source: ISME J. 2023 Sep 18;17(11):2070–8. doi: 10.1038/s41396-023-01511-z (PMC10579250; doi:10.1038/s41396-023-01511-z)
Supplement: Supplementary file 1 — SUP [file 41396_2023_1511_MOESM1_ESM.pdf]

Supplementary information – Additional figures and tables

"***Single cell dynamics and nitrogen transformations in the chain forming diatom Chaetoceros affinis***"

Authors: Rickard Stenow[1], Elizabeth K. Robertson[1], Martin J. Whitehouse[2] and Helle Ploug[1]

[1]Department of Marine Sciences, University of Gothenburg, Box 461, SE 405 30 Gothenburg, Sweden

[2]Swedish Museum of Natural History, Box 50 007, SE 104 05 Stockholm, Sweden

Supplementary figures

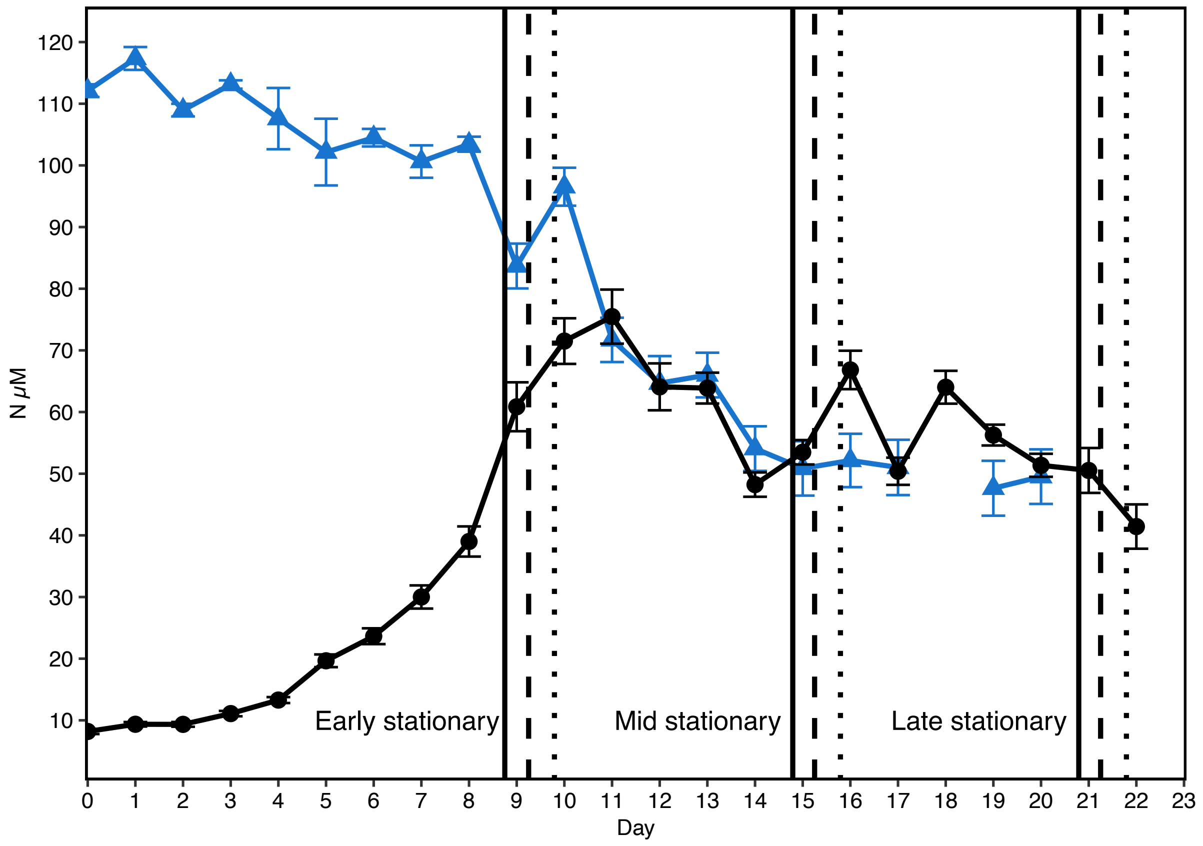

Fig. S1 NO_3^- concentration (blue triangles), PON estimated from plate reader data continuously calibrated against manual cell count to account for changes in cellular pigment abundance (black circles). Vertical lines show sampling times after ^{13}DIC and $^{15}NO_3^-$: solid line: T0, dashed line: 12 h L and dotted line: 12:12 h L:D, for the early, mid, and late stationary growth phase, day 9, 15 and 21 respectively. Numbers on the x axis represent 15:00 during each day. The error bars represent SE.

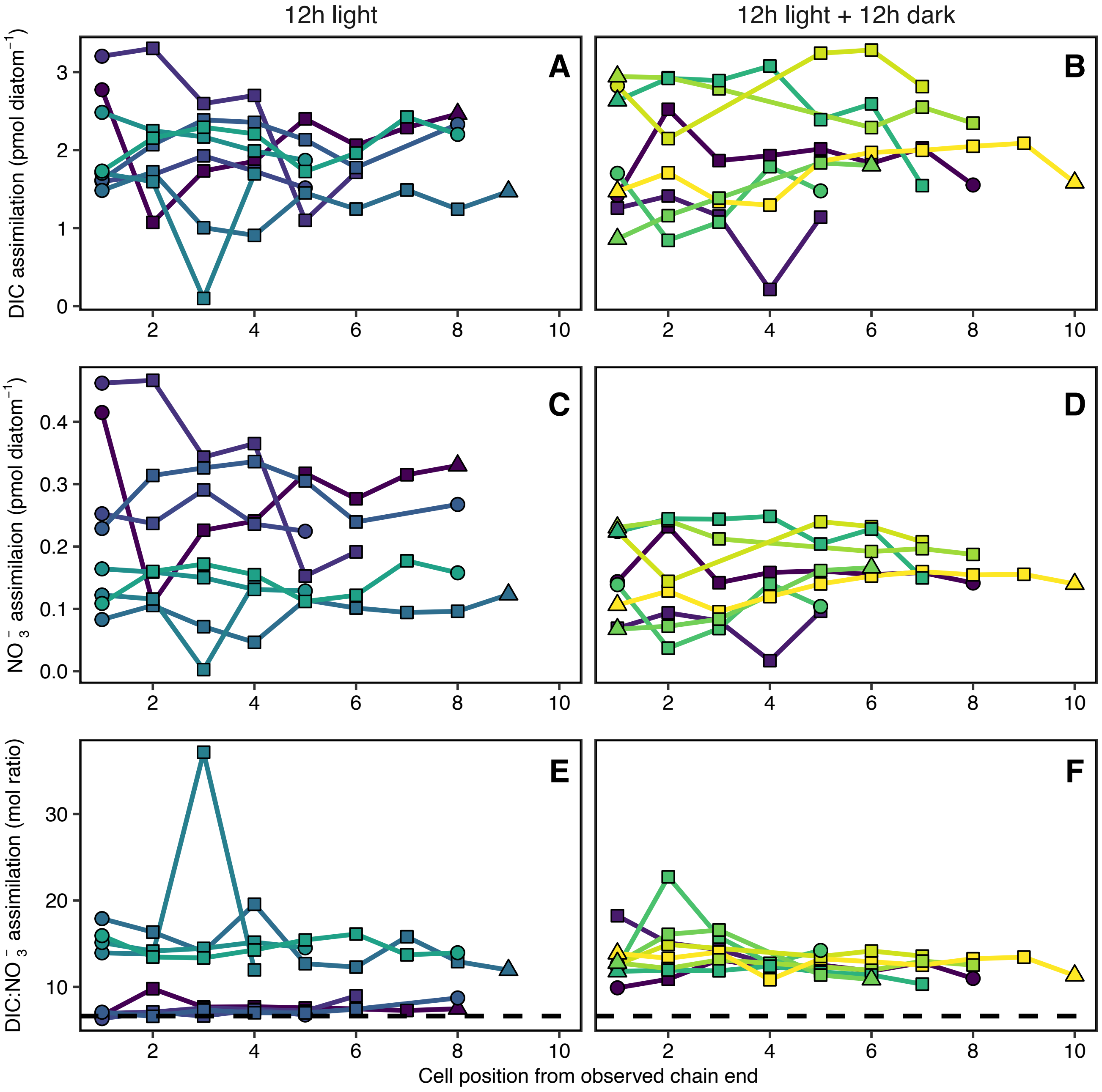

Fig. S2 DIC and NO_3^- assimilation by individual C. affinis cells in chains during the early stationary phase, as a function of the observed cells position relative to one end of the chain. Each line/color represents one chain, each symbol represents an individual cell, Circles: cells found at the end of a chain, Squares: cells not in the end of the chain, Triangles: cell position could not be determined, A, C & E: after 12h L, B, D & F: after 12:12h L:D, A & B: DIC assimilation by individual C. affinis cells in chains. C & D: NO_3^- assimilation by individual C. affinis cells in chains. E & F: DIC:NO_3^- assimilation ratio, dashed lines represent the Redfield C:N ratio.

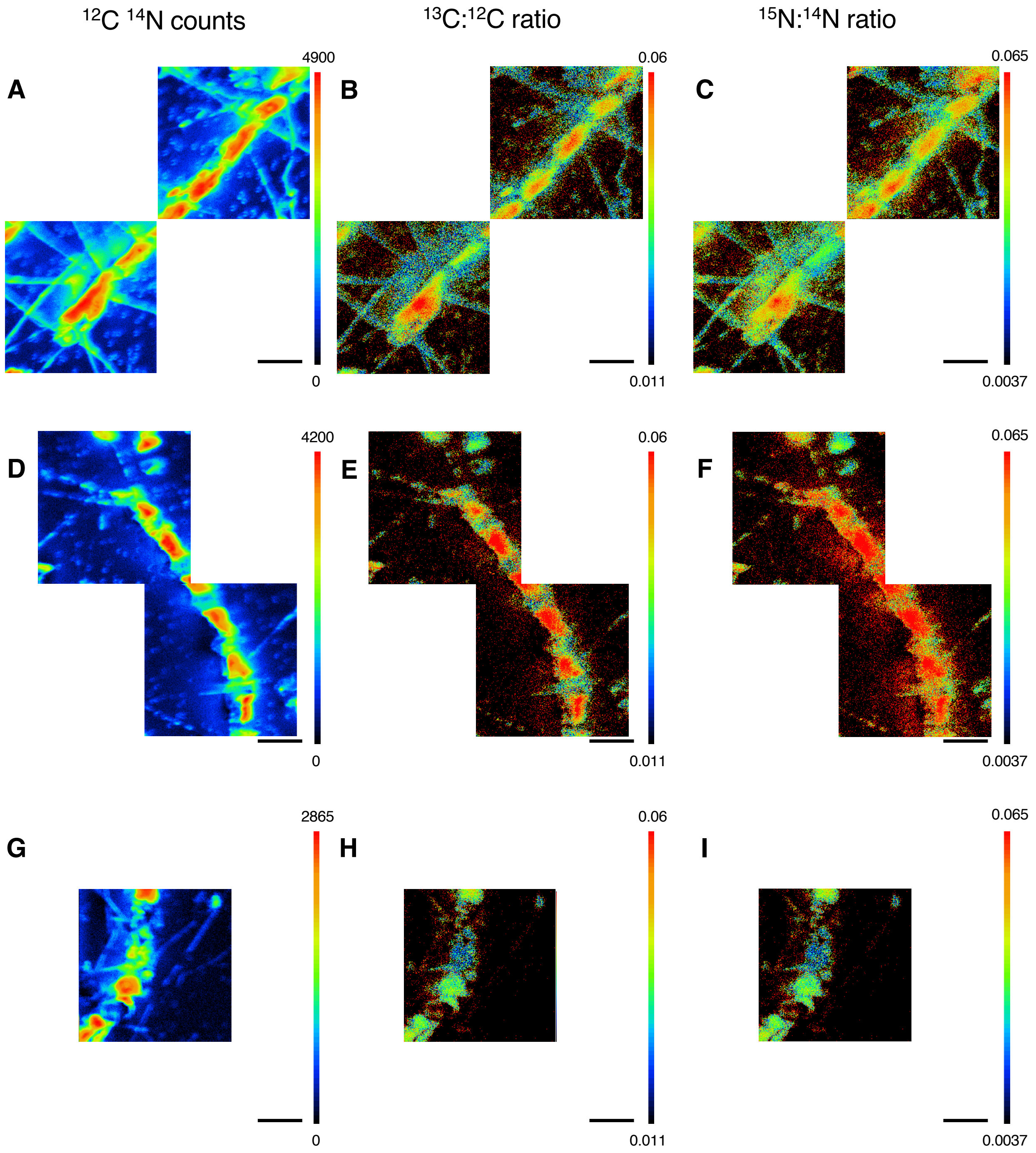

Fig. S3 DIC and NO_3^- assimilation in diatom chains, captured using SIMS. A-C: an active chain with one inactive cell (the second from the bottom left) after 12 h incubation in the early stationary growth phase, D-F: fully active chain in the early stationary growth phase after 24 h incubation, G-H: an active chain with a remineralized cell (second from the top) late stationary growth phase after 24h incubation, A, D, & G: $^{12}C^{14}N$ ion counts per pixel, a proxy for diatom biomass used to determine the location of each cell. B, E, & H: ^{13}C:^{12}C ratio, a proxy for DIC assimilation. C, F, & I: ^{15}N:^{14}N ratio, a proxy for NO_3^- assimilation. Scalebar represents 20 µm.

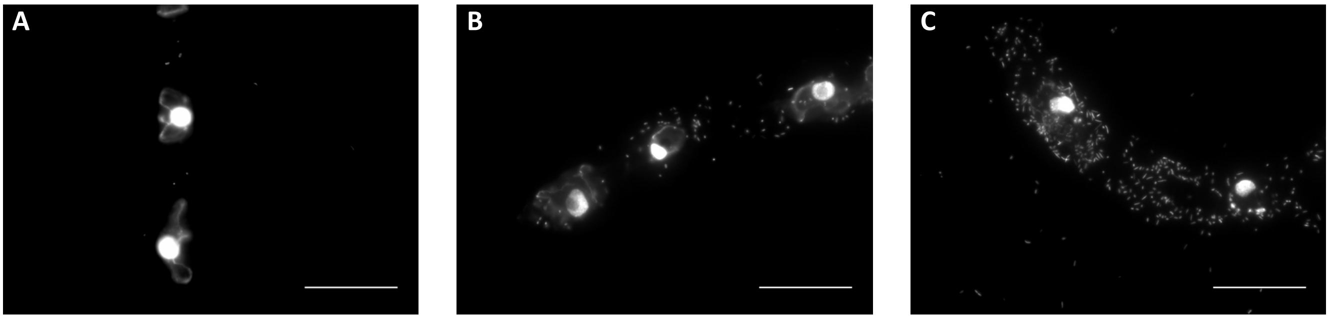

Fig. S4 Light microscopy images at 1000X showing abundance of DAPI stained diatom attached bacteria (small white dots) on the diatoms (lighter structures with a large white nucleus): A: early stationary phase, B: mid stationary phase and C: late stationary phase. The scale bar represents 20 µm.

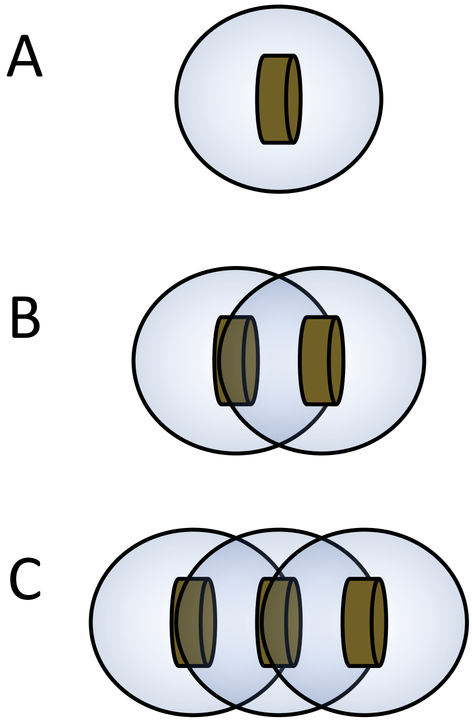

Fig. S5 A schematic view of diatoms (brown) and their diffusive boundary layer (blue). A: solitary cell, B: two cell chains, C: three cell chains. In two cell chains both cells experience similar diffusive fluxes (assuming a homogenous environment). While middle cells in three cell chains have more overlap of their boundary layers with neighboring cells and thus can expect lower diffusive fluxes to the cell compared to the end cells. Please note that size, shape, and proportions have been modified to better demonstrate how the diffusive boundary layers overlap varies with chain position.

Supplementary Tables

Table S1 Dissolved nutrient concentrations (average value ± SE for 3 replicates measured directly after isotopic tracer additions). The percentages represent NH_4^+ contribution to dissolved bioavailable DIN and the relative contribution to diatom assimilation in the last two columns respectively.

Growth phase	Dissolved Si (µM)	P (µM)	DIC (µM)	Dissolved NO_3^- (µM)	Dissolved NH_4^+ (µM)	Dissolved NH_4^+ (%)	Diatom NH_4^+:NO_3^- assimilation (%)
**Early stationary**	211 ±28	8.85 ±0.32	890 ±27	42.35 ±5.62	0.20 ±0.02	0.46%	n.d.
**Mid stationary**	194 ±45	6.71 ±1.09	815 ±22	38.92 ±8.90	0.35 ±0.04	0.88%	n.d.
**Late stationary**	139 ±24	7.98 ±0.22	792 ±7	27.98 ±4.72	1.59 ±0.20	5.37%	95.99%

Table S2 Assimilation of C and N by free-living and diatom attached bacteria, bacterial abundance, and transfer rates per diatom. Each value represents the mean ± SE.

Time	C assimilation free-living bacteria (fmol bacteria^{-1})	C assimilation diatom attached bacteria (fmol bacteria^{-1})	N assimilation free-living bacteria (fmol bacteria^{-1})	N assimilation diatom attached bacteria (fmol bacteria^{-1})	Attached bacteria per diatom (bacteria diatom^{-1})	C transferred per diatom (fmol diatom^{-1})	N transferred per diatom (fmol diatom^{-1})	C assimilation per diatom (fmol diatom^{-1})	N assimilation per diatom (fmol diatom^{-1})	C transferred to bacteria compared to diatom assimilation (%)	N transferred to bacteria compared to diatom assimilation (%)
**Early stationary**											
12h L					5.23 ±0.13[a]			1 770 ±51[b]	114 ±3.71[b]		
12:12h L:D	0.17 ±0.05[c]	0.82 ±0.06[d]	0.085 ±0.015[c]	0.116 ±0.005[d]	10.06 ±0.42[e]	8.25 ±0.60	1.17 ±0.06	1 862 ±43[f]	141 ±3.82[f]	0.44	0.83
**Mid stationary**											
12h L	0.57 ±0.12[g]	0.59 ±0.06[h]	0.034 ±0.008[g]	0.038 ±0.003[h]	34.82 ±1.41[i]	20.54 ±2.09	1.32 ±0.11	653 ±48[j]	25.9 ±2.2[j]	3.15	5.09
12:12h L:D	0.13 ±0.03[k]	0.45 ±0.07[l]	0.009 ±0.002[k]	0.023 ±0.004[l]	35.97 ±4.07[m]	16.19 ± 2.52	0.83 ±0.14	652 ±36[n]	39.5 ±2.72[n]	2.48	2.10
**Late stationary**											
12h L	0.18 ±0.05[o]	0.07 ±0.02[p]	0.001 ±0.002[o]	0.001 ±0.001[p]	53.06 ±4.55[q]	3.71 ±1.06	0.05 ±0.06	461 ±56[r]	4.69 ±1.24[r]	0.80	1.07
12:12h L:D	0.07 ±0.02[s]	0.11 ±0.03[t]	-0.002 ±0.001[s]	0.000 ±0.001[t]	85.57 ±3.77[u]	9.41 ±2.61	0.01 ±0.08	484 ±38[v]	4.73 ±0.787[v]	1.94	0.11

Number of analyzed cells: [a]=749 bacteria on 143 diatoms, [b]=98 SIMS, [c]=21 nano-SIMS, [d]=121 nano-SIMS, [e]= 1086 bacteria on 109 diatoms, [f]=164 SIMS, [g]=22 nano-SIMS, [h]=102 nano-SIMS, [i]=2 284 bacteria on 66 diatoms, [j]=50 SIMS, [k]=43 nano-SIMS, [l]=60 nano-SIMS, [m]= 2 519 bacteria on 69 diatoms, [n]=110 SIMS, [o]=17 nano-SIMS, [p]=47 nano-SIMS, [q]=2 749 bacteria on 53 diatoms, [r]=71 SIMS, [s]=56 nano-SIMS, [t]=110 nano-SIMS, [u]=3 148 bacteria on 37 diatoms and [v]=73 SIMS

Table S3 Diatom abundance, POC, PON, total DIC and DIN assimilation and DIC and DIN assimilation measured in C. affinis by SIMS. Each value represents the mean value, ± SE. Assimilation from T0 until sampling (12h L, 12h D or 12:12h L:D) assimilation for 12h D was derived as the difference in assimilation between 12h L and 12:12h L:D. Note that total assimilation rates include C. affinis and bacteria, while C. affinis assimilation rates were derived from cell-specific measurements C. affinis multiplied by diatom cell abundance.

Time	*C. affinis* abundance (cells mL^{-1})	POC (µM)	PON (µM)	Total DIC assimilation (µM)	Total NO_3^- assimilation (µM)	DIC assimilation *C. affinis* (µM)	NO_3^- assimilation *C. affinis* (µM)	NH_4^+ assimilation *C. affinis* (µM)	POC:PON (mol ratio)	DIC:DIN total assimilation (mol ratio)	DIC: NO_3^- *C. affinis* assimilation (mol ratio)
**Early stationary**											
T0	26 900 ±697[a]	214 ±27[b*†]	30 ±4[b*†]						7.1		
12h L	31 200 ±6 310[c]	410 ±1[b*]	53 ±2[b*]	64.9 ±4.2[b]	4.04 ±0.17[b]	41 ±1[d]	2.63 ±0.09[d]		7.3	16.1	15.1
12h D				5 ±5.7	0.39 ±0.40	0 ±2[▲]	0.45 ±0.16[▲]				
12:12h L:D	23 500 ±1 610[e]	415 ±29[b†]	60 ±4[b†]	65.0 ±1.4[b]	4.43 ±0.23[b]	41 ±1[f]	3.08 ±0.07[f]		7.0	14.7	13.2
**Mid stationary**											
T0	50 200 ±11 800[g]	328 ±41[b]	49 ±7[b]						6.7		
12h L	38 500 ±2 680[h]	401 ±120[b]	43 ±6[b]	49.2 ±6.2[b]	3.04 ±0.80[b]	23 ±2[i]	0.90 ±0.08[i]		9.2	18.9	25.2
12h D				-2.3 ±7.2	-0.75 ±1.19	1 ±3	0.58 ±0.18[▲]				
12:12h L:D	34 400 ±1 600[j]	351 ±37[b]	47 ±4[b]	46.9 ±1.0[b]	2.29 ±0.39[b]	24 ±1[k]	1.48 ±0.10[k]		7.5	20.5	16.5
**Late stationary**											
T0	25 300 ±1 240[l]	328 ±12[b]	43 ±2[b]						7.6		
12h L	34 400 ±3 230[m]	362 ±23[b]	47 ±4[b]	24.6 ±2.7[b]	0.98 ±0.70[b]	14 ±2[n]	0.15 ±0.04[n]	2.87 ±0.24[o]	7.6	25.2	97.9
12h D				-8.4 ±3.6[▲]	-0.61 ±0.74	-1 ±3	0.02 ±0.06				
12:12h L:D	25 200 ±1 430[p]	342 ±11[b]	45 ±1[b]	16.2 ±0.9[b]	0.37 ±0.04[b]	13 ±1[q]	0.13 ±0.02[q]		7.6	44	102

Number of analyzed cells: [a]=927 diatoms, [b]=N=3 EA-IRMS, [c]=994 diatoms, [d]=98 SIMS, [e]=919 diatoms, [f]=164 SIMS, [g]=943 diatoms, [h]=915 diatoms, [i]=50 SIMS, [j]=926 diatoms, [k]=110 SIMS, [l]=944 diatoms, [m]=935 diatoms, [n]=71 SIMS, [o]=73 SIMS, [p]=927 diatoms, [q]=93 SIMS, Significant difference t-test. p>0.05: [*]=T0 vs Light 12h, [▲]=Light 12h vs L 12h+D 12h and [†]=T0 vs L 12h+D 12h

Table S4 Average chain length and cell size during each growth phase ± SE, n = 9. *Observations were made using light microscopy.*

**Time**	**Chain length (diatom cells chain^{-1})**	**Length (µm)**	**Width (µm)**	**Volume (µm^3)**	**Surface area (µm^2)**	**Area:Volume**
**Early stationary**	5.20 ±0.13	22 ±0.22	12 ±0.14	2500	1100	0.43
**Mid stationary**	3.47 ±0.09	24 ±0.28	10 ±0.19	2000	940	0.48
**Late stationary**	2.56 ±0.08	26 ±0.22	11 ±0.18	2500	1100	0.44

Table S5 Measured C and N content cell^{-1} and estimated content using Redfield 1934, Menden-Deuer and Lessard 2000, Sun 2003. During the early mid and late stationary growth phase here shortened to E-STAT, M-STAT, and L-STAT respectively. Each value represents means ± SD. Bold values are averages for the entire incubation.

Time	Measured C content (pg cell^{-1})	Estimated C content (pg cell^{-1})	Measured N content (pg cell^{-1})	Estimated N content (pg cell^{-1})
**E-STAT**	**167 ±68**	**159**	**27 ±11**	**28**
T0	96 ±21	154	16 ±4	27
12 h L	199 ±22	163	30 ±1	29
12:12 h L:D	216 ±55	159	36 ±9	28
**M-STAT**	**111 ±39**	**133**	**17 ±5**	**23**
T0	89 ±40	144	15 ±7	25
12 h L	121 ±48	124	16 ±3	22
12:12 h L:D	124 ±31	130	19 ±4	23
**L-STAT**	**149 ±21**	**161**	**23 ±4**	**28**
T0	156 ±12	156	24 ±2	27
12 h L	127 ±11	175	19 ±2	31
12:12 h L:D	164 ±17	152	25 ±4	27
**L-STAT NH_4^+**	**145 ±19**	**150**	**21 ±3**	**26**
T0	150 ±15	138	23 ±3	24
1.5 h L	138 ±20	152	20 ±3	27
4.15 h L	146 ±26	159	21 ±3	28

Table S6 A compilation of measurements, shown as percentages (%) relative to the value in the early stationary growth phase.

Measurement	Early stationary	Mid stationary	Late stationary
Dissolved nutrients			
DIC (μM)	100	92	89
NO_3^- (μM)	100	93	67
NH_4^+ (μM)	100	175	795
P (μM)	100	76	90
Si (μM)	100	92	66
Particulate organic matter			
POC (μM)	100	98	88
PON (μM)	100	81	89
Diatom abundance and properties			
Abundance (diatoms mL^{-1})	100	123	110
Chain length (diatom cells $chain^{-1}$)	100	69	49
Measured C content (pg C $diatom^{-1}$)	100	66	89
Estimated C content (pg C $diatom^{-1}$)	100	84	101
Measured N content (pg N $diatom^{-1}$)	100	63	85
Estimated N content (pg N $diatom^{-1}$)	100	82	100
Proportion of active cells among solitary diatoms	100	78	58
Proportion of active diatoms in chains	100	87	48
Diatom C and N assimilation in solitary cells			
DIC assimilation active+inactive (pmol $diatom^{-1}$)	100	38	24
DIC assimilation only active (pmol $diatom^{-1}$)	100	46	39
NO_3^- assimilation active+inactive (pmol $diatom^{-1}$)	100	32	4
NO_3^- assimilation only active (pmol $diatom^{-1}$)	100	40	6
Diatom C and N assimilation in chains			
DIC assimilation active+inactive diatoms (pmol $diatom^{-1}$)	100	35	27
DIC assimilation only active diatoms (pmol $diatom^{-1}$)	100	39	39
NO_3^- assimilation active+inactive diatoms (pmol $diatom^{-1}$)	100	25	4
NO_3^- assimilation only active diatoms (pmol $diatom^{-1}$)	100	28	5
Diatom attached bacteria, abundance, assimilation of diatom derived C and N			
Attached bacteria per diatom (bacteria $diatom^{-1}$)	100	666	1015
Diatom derived ^{13}C-DIC transferred to attached bacteria (fmol $bacteria^{-1}$)	100	55	13
Diatom derived ^{15}N-NO_3^- transferred to attached bacteria (fmol $bacteria^{-1}$)	100	20	0
^{13}C transferred to bacteria per diatom (fmol $diatom^{-1}$)	100	196	114
^{15}N transferred to bacteria per diatom (fmol $diatom^{-1}$)	100	71	1
Free living bacteria, assimilation of diatom derived C and N			
Diatom derived ^{13}C-DIC transferred to free-living bacteria (fmol $bacteria^{-1}$)	100	76	41
Diatom derived ^{15}N-NO_3^- transferred to free-living bacteria (fmol $bacteria^{-1}$)	100	11	0

Table S7 C- and N-specific growth rates for diatom attached and free-living bacteria. Each value represents the mean value ± SE. Numbers in brackets represents the highest and lowest positive observation.

Time	C specific growth rate free-living bacteria (h^{-1})	C specific growth rate diatom attached bacteria (h^{-1})	N specific growth rate free-living bacteria (h^{-1})	N specific growth rate diatom attached bacteria (h^{-1})
**Early stationary**				
12:12h L:D	1.01E-03 ±2.98E-04[a] (0 – 6.28E-03)	4.77E-03 ±3.38E-04[b] (0 – 1.34E-02)	2.52E-03 ±4.59E-04[a] (0 – 6.99E-03)	3.45E-03 ±1.59E-04[b] (0 – 7.02E-03)
**Mid stationary**				
12h L	6.61E-03 ±1.39E-03[c] (0 – 1.57E-02)	6.85E-03 ±7.38E-04[d] (0 – 2.57E-02)	2.00E-03 ±4.73E-04[c] (0 – 6.04E-03)	2.28E-03 ±1.87E-04[d] (0 – 7.98E-03)
12:12h L:D	7.29E-04 ±1.53E-04[e] (0 – 5.60E-03)	2.60E-03 ±4.29E-04[f] (0 – 1.09E-02)	2.60E-04 ±5.09E-05[e] (0 – 1.78E-03)	6.96E-04 ±1.15E-04[f] (0 – 3.07E-03)
**Late stationary**				
12h L	2.15E-03 ±5.83E-04[g] (0 – 8.86E-03)	7.68E-04 ±2.16E-04[h] (0 – 7.95E-03)	6.34E-05 ±9.99E-05[g] (0 – 8.64E-04)	6.52E-05 ±6.69E-05[h] (0 – 1.08E-03)
12:12h L:D	4.20E-04 ±1.34E-04[i] (0 – 7.06E-03)	6.57E-04 ±1.54E-04[j] (0 – 9.51E-03)	-6.99E-05 ±4.33E-05[i] (0 – 6.52E-04)	2.23E-06 ±2.74E-05[j] (0 – 5.52E-04)

Number of replicates: [a]=N=21 nano-SIMS, [b]=121 nano-SIMS, [c]=22 nano-SIMS, [d]=102 nano-SIMS, [e]=43 nano-SIMS, [f]=60 nano-SIMS, [g]=17 nano-SIMS, [h]=47 nano-SIMS, [i]=56 nano-SIMS and [j]=110 nano-SIMS
